# Targeted *de novo* phasing and long-range assembly by template mutagenesis

**DOI:** 10.1093/nar/gkac592

**Published:** 2022-07-13

**Authors:** Siran Li, Sarah Park, Catherine Ye, Cassidy Danyko, Matthew Wroten, Peter Andrews, Michael Wigler, Dan Levy

**Affiliations:** Cold Spring Harbor Laboratory, Cold Spring Harbor, NY 11724, USA; Cold Spring Harbor Laboratory, Cold Spring Harbor, NY 11724, USA; Cold Spring Harbor Laboratory, Cold Spring Harbor, NY 11724, USA; Cold Spring Harbor Laboratory, Cold Spring Harbor, NY 11724, USA; Cold Spring Harbor Laboratory, Cold Spring Harbor, NY 11724, USA; Cold Spring Harbor Laboratory, Cold Spring Harbor, NY 11724, USA; Cold Spring Harbor Laboratory, Cold Spring Harbor, NY 11724, USA; Cold Spring Harbor Laboratory, Cold Spring Harbor, NY 11724, USA

## Abstract

Short-read sequencers provide highly accurate reads at very low cost. Unfortunately, short reads are often inadequate for important applications such as assembly in complex regions or phasing across distant heterozygous sites. In this study, we describe novel bench protocols and algorithms to obtain haplotype-phased sequence assemblies with ultra-low error for regions 10 kb and longer using short reads only. We accomplish this by imprinting each template strand from a target region with a dense and unique mutation pattern. The mutation process randomly and independently converts ∼50% of cytosines to uracils. Sequencing libraries are made from both mutated and unmutated templates. Using de Bruijn graphs and paired-end read information, we assemble each mutated template and use the unmutated library to correct the mutated bases. Templates are partitioned into two or more haplotypes, and the final haplotypes are assembled and corrected for residual template mutations and PCR errors. With sufficient template coverage, the final assemblies have per-base error rates below 10^–9^. We demonstrate this method on a four-member nuclear family, correctly assembling and phasing three genomic intervals, including the highly polymorphic HLA-B gene.

## INTRODUCTION

Third-generation sequencing platforms such as PacBio and Oxford Nanopore generate long-range sequencing information useful for high-quality genome assemblies (1,2). Long-reads are especially important when the genome studied is diploid in order to phase distant heterozygous sites (3,4). These platforms are error-prone and costly, particularly when compared to the present generation of short-read sequencing machines. Short-read sequencers, however, cannot phase distant variants or assemble genomic regions with complex repeats.

We previously introduced muSeq (5,6), a method for obtaining long-read information from short-reads by first marking each template with a unique mutational pattern. Mutation patterns were generated by partial bisulfite conversion, randomly deaminating ∼50% of cytosine positions, which converts **C** to **T** in the resulting sequence libraries. By identifying reads with matching or overlapping mutational patterns, we were able to count template molecules and assemble mapped cDNA reads into full-length isoforms. The previous muSeq informatics relied on mapping reads to a reference genome. This limited its utility to well-sequenced organisms. Even in those cases, muSeq would struggle to resolve insertion/deletion polymorphisms, splice junctions, and complex genomic regions rich in polymorphisms such as the HLA locus. It also struggled in long template preservation and amplification.

In this paper, we remove the need for a reference genome, presenting two bench protocols and a computer program for haplotype-phased de novo assembly using muSeq. The new protocols have significant improvements in template length. Both bench protocols combine targeted, long-range amplification with template mutagenesis. In the first protocol, we use partial bisulfite conversion to introduce random mutation patterns into the amplified templates. In the second protocol, we remove the need for chemical mutagenesis by randomly incorporating methyl-cytosines into copies of the initial templates. We then convert the unmethylated cytosines using an enzymatic deaminase generating mutated templates greater than 10 kb in length. Both methods generate sequencing libraries from between 50 and 1000 unique full-length mutated templates.

The computer program we present in this paper takes these sequencing libraries as inputs and returns a set of haplotype sequences. The program first disentangles the mutated template molecules using simple and conservative de Bruijn graph assembly methods, which are then augmented with paired-end read information. In lieu of a reference genome, the program uses an unmutated sequence library to correct mutations introduced by the deamination process. The program then identifies a collection of haplotypes that best explain the observed data. For a diploid region, this divides the templates into two sets. In the final steps, the program takes a consensus sequence for each of the two sets, eliminating PCR errors and residual mutations. When complete, the program returns two haplotype sequences.

To demonstrate the protocol and informatics, we targeted two 5 kb genomic regions and one 10 kb region using DNAs derived from a four-member nuclear family (father, mother, and two children) with prior whole-genome sequence data. One region was selected for its difficulty to phase (>1 kb stretches of homozygosity) and the second for its difficulty to map (HLA). The resulting muSeq *de novo* assemblies over both regions precisely recapitulate the sequence results in the parents as expected by inheritance and in agreement with the WGS data. Additionally, *in silico* read-mixing experiments confirm that the algorithm accurately disentangles mutated templates and can resolve complex cases with more than two haplotypes. Finally, we exceeded our upper limit for target size by switching to a new protocol, capable of encoding mutation patterns in templates greater than 10 kb in length.

## MATERIALS AND METHODS

**Figure 1.**
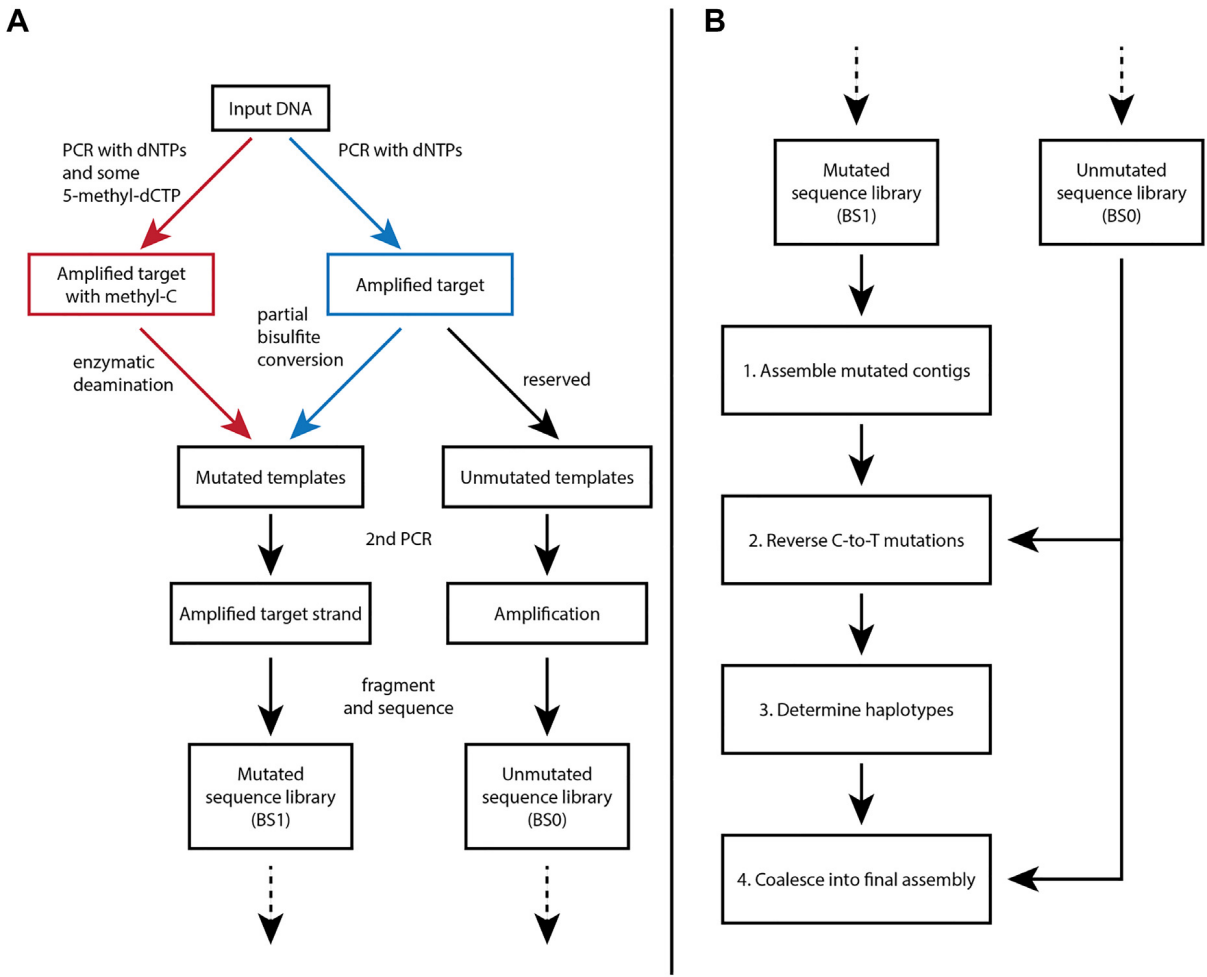
muSeq workflow diagram. Panel **A** shows the bench component of the protocol while panel **B** shows the informatics. There are two methods for obtaining mutated templates: along the blue arrows, we first amplify the target region and then perform a partial bisulfite reaction; along the red arrows, we first amplify the target region with a mixture of dNTPs that also contain 5-methyl-dCTP. The resulting templates contain a mixture of C and methyl-C, so that after complete enzymatic deamination, we obtain mutated templates similar to those obtained through bisulfite treatment. The mutated templates are then selectively amplified for one of the two strands, and fragmented into a sequencing library. An unmutated portion of the initial template is also reserved to generate an unmutated sequencing library. Panel B begins with the sequencing reads from the two libraries and outlines the major steps in the assembly pipeline. (1) Mutated reads are used to assemble mutated contigs, then (2) we use the unmutated library to reverse as many of the C-to-T mutations as possible. From the collection of templates over the region, we (3) determine the haplotypes and with reference to the unmutated reads (4) we coalesce into a final assembly for each haplotype complete with quality score.

The muSeq method has two primary components: wet-bench protocols that imprint template molecules with a mutation pattern, and an informatics protocol that assembles haplotype sequences (Figure 1). We have developed two bench protocols: the first protocol uses sodium bisulfite and can target regions up to 5 kb in length. The second protocol randomly incorporates methyl-cytosines and uses enzymatic deamination to target regions 10 kb and longer. In practice, we amplified from 50 to 1000 mutated templates, which are then randomly fragmented into Illumina sequencing libraries. The informatics pipeline uses the short-read data to assemble as many of those mutated templates as possible. Each mutated template is assembled into a long sequence derived from a single haplotype. We use this long-range information to resolve SNV divergence between the haplotypes, coalescing sequences from many unique templates into haplotype-specific assemblies.

We obtained DNA from a four-member nuclear family with prior whole-genome sequence data, comprising a mother, a father, and two children (identified as ‘proband’ and ‘sibling’). Over most 5–10 kb regions of the genome, the mother will have two distinct haplotypes (A and B), the father will have two distinct haplotypes (C and D), and, excluding recombination and *de novo* mutation, each child will inherit either A or B from the mother and either C or D from the father. This provides a ‘built-in check’ for our haplotype assembly method.

In total, we targeted three regions: Region 1 (chr14:92426797–92432250) was selected as a region with few variants separated by long blocks of homozygosity. Region 2 (chr6:31352646–31358270), containing the entire HLA-B gene, was selected as a region dense in variation with poor mapping to the reference genome. Region 3 (chr18:11666735–11677605) was selected as a 10 kb span to test our protocol for enzymatic mutagenesis.

### Protocol I: Bisulfite mutagenesis

To prepare templates for bisulfite mutation, we first amplify a muSeq target region by PCR (Figure 1A). We reserve one aliquot of the PCR products for an unconverted library while we subject a second aliquot to partial bisulfite conversion with conditions optimized to randomly convert 40–60% of cytosines. We then apply a second round of PCR using a nested primer pair. The primer target sites are selected to be largely resistant to mutation in one of the two strands. Samples were sonicated to an average length of 400 bp and prepared for standard Illumina sequencing. Both converted and unconverted libraries were sequenced on a MiSeq in 150 bp paired-end mode at a median depth of one million read-pairs per individual per region.

### Protocol II: enzymatic mutagenesis

A new method for assessing genome-wide DNA methylation uses enzymatic protection of methyl-cytosines followed by enzymatic deamination of unprotected cytosines using an APOBEC enzyme (7). As enzymatic treatment is less likely to fragment DNA than bisulfite treatment, we sought to harness it for partial conversion. However, APOBEC is a processive enzyme and thus is not suitable for random deamination (8). Instead, we introduce randomness by amplifying the target sequence with a mixture of dNTPs that include 5-methyl-dCTP at a 1:1 ratio of dCTP to 5-methyl-dCTP in the first round of PCR (Figure 1A). This mixture results in the random incorporation of methyl-C into the copies of the template molecules at a rate of about 40–60% (see Supplementary Methods for more details). Subsequently, the methyl-C nucleotides were oxidized by TET2 into 5-hydroxymethylcytosine, leaving only the un-methylated dCTPs susceptible to the next step, C-to-U deamination by APOBEC. After the APOBEC mutation, we perform a second round of PCR as with Protocol I. Unexpectedly, we found that including 5-methyl-dCTP in the PCR nucleotide mixture significantly improved the success rate of getting full-length products. We do not know the reason for this. With the second PCR product, we proceeded with sonication and sequencing library preparation as above.

### Computational pipeline

Generating haplotype sequence assemblies from the mutated and unmutated sequence reads requires a multi-step computational pipeline (Figure 1B). First, we assemble mutated template sequences using de Bruijn graphs augmented with paired-end read information. Next, we ‘unmask’ most of the mutated positions to their prior base by using a lookup table built from the unconverted sequence library. After unmasking, we co-align the template sequences, relying on heterozygous SNVs to determine a parsimonious set of haplotypes. Finally, we aggregate templates from the same haplotype into a consensus sequence, resolving still-masked polymorphism, and assigning a quality score for every position. We provide an overview of the method steps below and include details of the implementation in the Supplementary Methods. We also include a link to commented source code.

#### Step one: assembling contigs of mutated templates

The first step in our computational pipeline assembles long mutated contigs, each derived from one mutated template. There are two key parameters in this process: ***k***, the length of the *k*-mer used for initial assembly, and *D*, the disruption parameter which reflects the tolerance for mismatch in the pair-end extension process. To establish initial contigs, we use the mutated reads to construct a de Bruijn graph from subsequences of length *k* (*k*-mers) and identify unambiguous paths. The choice of *k*-mer length strongly determines the distribution of initial contig lengths. Longer *k*-mers have greater sequence diversity, reducing the chance that two distinct mutated templates share a common *k*-mer. However, longer *k*-mers require higher sequence coverage to obtain uninterrupted paths. By counting the number of distinct mutation patterns for each unmutated *k*-mer, we can estimate the number of original starting templates. The number of full-length initial contigs rarely reflect the number of starting templates. To greatly improve the yield of full-length contigs, we employ paired-end reads. The procedure for joining initial contigs into extended contigs, detailed in the supplement, strongly depends on the parameter *D*, which controls for how much single-base amplification error is tolerable when extending a contig.

**Figure 2.**
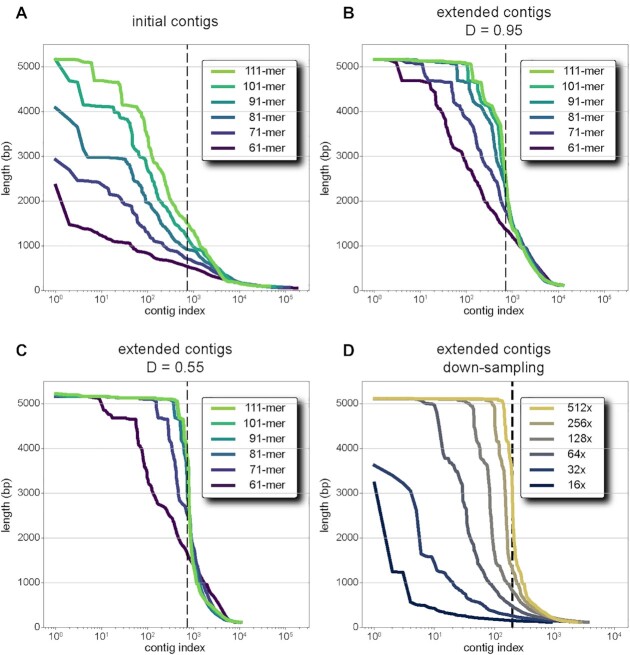
Contig length distributions. Each panel shows the distribution of contig lengths, sorted from longest to shortest with a log-scale for the x-axis. Panel **A**–**C** show data derived from *in silico* mixing of eight libraries, four individuals and both strands, from region 1 for various choices of *k*-mer size (line color). The dashed black line at 725 shows the expected number of templates (see Supplementary Figure S1). Panel A shows the sorted distribution of initial contig lengths from the de Bruijn graph. In the next assembly step, we use paired-read information to join initial contigs. This is subject to a disruption parameter, (**D**) Panels B and C show the sorted distributions of extended contig lengths when the disruption parameter is restrictive (panel B, 0.95) and less restrictive (panel C, 0.55). Panel D shows extended contig lengths from one library subject to down-sampling. The dashed black line at 200 indicates the expected number of templates. The color of the line indicates the number of reads used in terms of redundant coverage per-template. The maximum coverage of 512× per template amounts to 1.74 million 150 bp paired-end reads while the minimum coverage of 16x per template amounts to 54 000 paired-end reads. At 128× per-template coverage or ∼400 000 reads, we recover ∼50 full-length templates.

To illustrate this process, we explore the distribution of contig length for the 5 kb region 1, using data from both strands and all four individuals, eight libraries in total. By counting the number of distinct mutation patterns for each unmutated *k*-mer, we estimate that there are ∼725 original templates, approximately 100 templates per strand per person (see Supplementary Figure S1). In Figure 2A, we show the distribution of initial contig lengths as a sorted plot for a range of *k*-mer length, with the estimated total of 725 templates shown as a dotted vertical line. Using 61-mers, the longest initial contig is shorter than 2.5 kb. However, with 111-mers, the de Bruijn graph yields between 10 and 20 full-length initial contigs.

To get many more long contig assemblies, we use paired-end reads to join initial contigs into extended contigs. The performance of extension depends upon the parameter *D*, and we explore a range of values. For the highest value (0.95) most intolerant of base-error, even the 111-mers return only 100 full length initial templates (Figure 2B). While at the lowest value (0.55), we recover better than 90% of the full-length templates (Figure 2C). We were concerned that increasing tolerance to PCR error might lead to assembly of chimeric contigs. To monitor this, we tracked every read-pair to the individual and the strand from which it arose. Happily, we found that for every value of *D*, all full-length contigs derive their reads from one individual and one strand. For Region 3, we adapt this method, by making two separate libraries from the same strand and monitoring for chimerism.

#### Step two: unmasking mutations

The extended contigs still bear their mutation patterns with **T** at many positions that initially were **C**. In the second step, we use the unmutated library to ‘unmask’ the mutation patterns from the extended contigs, restoring the **C** at most (but not all, see below) mutated positions. To do this, we map unmutated *k*-mers from the unmutated library to the mutated contigs. An unmutated *k*-mer is properly mapped if the aligned sequence in the mutated contig could have arisen from the unmutated *k*-mer by **C**-to-**T** conversions. About two-thirds of the time, the pileup of unmutated *k*-mers over a **T** position confidently reports a **T**, and about a third of the time, the pileup confidently reports a **C** (better than 99%). In both cases, we unmask the **T** in the contig to a **C** or a **T** according to the pileup consensus. In a handful of cases, the pileup is split between **C** and **T**. This happens when the position is heterozygous or repeated imperfectly elsewhere in the region. We do not unmask such positions at this time, resolving them only in step 4, after templates are partitioned into haplotypes.

#### Step three: partitioning haplotypes

When the unmasked contigs are all derived from the same haplotype, they will co-align exactly except for the unresolved **T** positions and early template amplification errors. However, when the contig sequences derive from two or more haplotypes, SNVs and indel differences between the haplotypes manifest as imperfect alignments between unmasked contigs. Over much of the genome, human haplotypes differ by ∼1 SNV per kb. We use the SNV differences between long assemblies of haplotypes to establish a parsimonious partition of the longer unmasked contigs (>1.5 kb) into distinct haplotypes.

We first select the longest unmasked contig to serve as a stand-in reference, and then use the Needleman-Wunch algorithm to co-align each of the longer unmasked contigs to this reference. We next summarize the mapped alignments, identifying bi-allelic positions and generating a matrix of observations: A row for each unmasked contig and a column for each bi-allelic position. A haplotype *H* is a base assignment for each bi-allelic position and, using a simple probabilistic model, we can determine the likelihood that each unmasked contig derived from haplotype *H*. We can naturally extend this likelihood to pairs of haplotypes (*H_1_* and *H_2_*) or any ploidy *M* (*H_1_* … *H_M_*) by setting the likelihood that an unmasked contig derives from a set of haplotypes as the maximum probability that it derived from any one of them. If the number of bi-allelic positions and haplotypes is small, we compute the probability over all possible configurations to identify the global maxima. Otherwise, we apply a simulated annealing algorithm to search for the optimal solution. Details of how to identify SNVs, express the likelihood function, and apply simulated annealing are included in the Supplementary Methods and extensively commented code. At the end of the process, we build a set for each haplotype comprised of the unmasked contigs that are far more likely (better than 1 in a 1000) to derive from that haplotype than any of the others.

#### Step four: final alignment

In the fourth and final step, contigs from the same haplotype are co-aligned and averaged to eliminate residual mutation (e.g. at C–T polymorphisms) and PCR errors. Alignment proceeds as before, using the Needleman-Wunch algorithm and starting from the longest unmasked contig in a haplotype set. However, to account for the chance of encountering an indel error in the longest contig, we include an iterative loop that corrects for indels errors. After alignment, for each position in the common alignment, we summarize the base calls in all contigs that cover that position. Finally, we report the most likely base under the same probabilistic model used in step three. One consequence of the model is the assignment of a confidence score to each base in the assembly. For positions that are covered by at least 10 templates, the error rates are typically in the range of 10^–9^. We log-transform this probability into a Phred quality score reporting the set of sequences and qualities in a FASTQ output file.

## RESULTS

### Experimental design

Previously, we established the theory of sequence assembly and enumeration from partially mutated templates (5). We then demonstrated feasibility for short DNA and cDNA fragments given actual bench protocols utilizing a reference sequence to facilitate read alignment (6). In the following, we sought to assemble long fragments of DNA without a reference sequence. Moreover, we sought to achieve this from a diploid genome for which assembly is confounded by haplotypic sequence variation. For this purpose, we initially chose two regions of the human genome: Region 1 with a low incidence of variation and Region 2 with exceptionally high sequence variation encompassing the HLA-B gene. In these two cases, we used bisulfite mutagenesis for partial deamination. Bisulfite mutagenesis is harsh and degrades DNA, thus limiting the size of the region we could mutagenize to ∼5 kb. During the course of our work, we developed a new method for partial mutagenesis by randomly incorporating methylated cytosine into copies of the templates prior to complete enzymatic deamination. As this method is gentle, we tested this protocol on the larger 10 kb Region 3.

Assembly from a library of long mutated templates, in the absence of a reference map, required the development of new algorithms and new methods for validation. For *de novo* assembly of the mutated templates, we use de Bruin graphs to build initial contigs and then use paired-end reads to join initial contigs into extended contigs. Each extended contig derives from a single initial template still imprinted with its unique mutation pattern. To restore the initial sequence to its pre-mutated state, we use *k*-mer counts obtained from an unmutated library over the same region to ‘unmask’ positions that were certainly **C** before mutation to **T**. Positions that cannot be unmasked this way are either **C**/**T** heterozygous sites or duplicated regions with **C**/**T** variants. Although few in number, they are important for haplotypic assembly and resolving duplication. These ambiguous positions are resolved after the haplotypes are settled. We next partition the unmasked contigs into haplotype-specific subsets using a maximum likelihood method. Within each haplotype subset, we resolve the still-masked heterozygous **C**/**T** site. This yields the final assemblies, with each position annotated by a quality score reflecting the confidence in the call.

To validate correct assembly, we compare to the reference map, and to validate haplotype resolution and variant resolution, we compare to the transmission patterns of the nuclear family obtained from WGS. We show that with sufficient template coverage, the confidence of assembled sequence is very high and that the algorithms are capable of resolving mixed genomes of high ploidy. Finally, we show how disentanglement of intentionally mixed libraries can be used to establish proper parameter settings for the algorithms.

### Region 1: locus with sparse variation

We begin our exploration of assembly using DNA from a four-member nuclear family with prior whole genome sequence data. For Region 1, we selected a 5 kb region (chr14:92 426 797–92 432 250) that has only a few variants separated by long blocks of homozygosity. The blocks are so long that phasing the variants in any individual is impossible using short sequence reads. However, because we have WGS from the four member nuclear family, we can infer the haplotypes and validate the results of muSeq.

**Figure 3.**
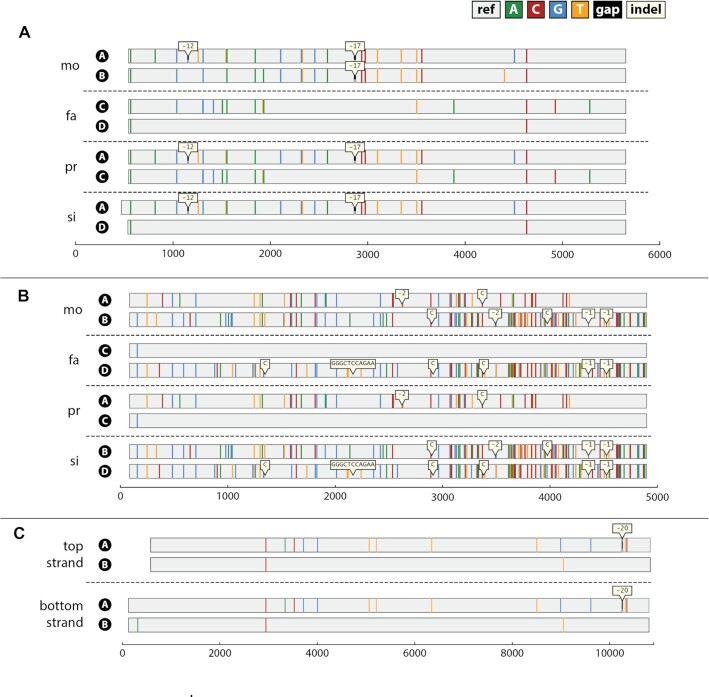
Assembly plots. A graphical description of the muSeq assemblies for each of the three regions (panels **A**–**C**). The haplotype assemblies are aligned to the reference genome sequence for the region with SNV differences depicted by color and indels shown in bubbles. Maternal haplotypes are labeled A and B; paternal haplotypes are labeled C and D. Panel A shows the final haplotype assemblies for region 1 for each of the four family members (mother, father, proband and sibling). The maternal haplotype A was inherited by both children, while paternal haplotype C is inherited by the proband and paternal haplotype D by the sibling. These results agree with the expectation from whole-genome sequencing. Panel B shows the final haplotype assemblies for region 2, which includes the HLA-B gene. As expected, the haplotypes are highly divergent. All but paternal haplotype C differ significantly from the reference genome. The parental haplotypes are in exact agreement with the children's haplotypes and each of the sequences has a perfect match to a known HLA-B variant in the IMGT/HLA genomic sequences database. Panel C shows the haplotype assemblies over a 10 kb region from the mother, using data from either the top strand or bottom strand. Both assemblies are in agreement and match expectations from whole-genome sequence data.

For this region, we generated two mutated libraries per family member: one targeting the top strand and one targeting the bottom strand. We first processed each family member separately using either (i) the top strand, (ii) the bottom strand or (iii) both (see Supplementary Table S1 for all haplo-assemblies). Using the top-stand mutation data and applying the assembly pipeline as described in the Materials and Methods, we obtained two full-length 5.1 kb contiguous assemblies per family member. These assemblies match the reference genome exactly except for the SNVs and deletions highlighted in Figure 3A. The figure depicts the haploid sequence assemblies from top-strand data for each member in the family. The haplotype sequence assemblies in the children are identical to assemblies found in the parents. Specifically, one maternal haplotype (A) is transmitted to both the proband and the sibling, while the other maternal haplotype (B) is transmitted to neither child. One paternal haplotype is transmitted to the proband (C) while the other is transmitted to the sibling (D). The full haplotype sequences, including 29 distinct SNVs and the two deletions, match the phasing inferred from the whole genome sequencing data for this family.

Our assembly algorithm assigns a quality score to each position, reflecting the confidence we have in the base-call. This confidence score is based on the base-call information from all templates covering that position. For our top-strand assemblies, all positions were covered by >40 templates per haplotype. Consequently, our base-call confidence was very high, with quality scores in excess of 90 (i.e. error less than 1 in 10^–9^). We sought to validate our top-strand assemblies with our bottom-strand assemblies. Here, we used fewer mutated templates, with some positions covered by fewer than 10 templates. This resulted in lower quality scores for these positions. The bottom-strand data confirmed the **C**/**T** heterozygous calls confidently made in the top-strand assemblies. However, at low-coverage, one **G**/**A** het locus (which appears as **C**/**T** in the bottom strand) had poor quality scores and an incorrect base-call (position 5276 in Supplementary Table S1). Since the **C**/**T** positions of one strand are **G/A** in the other, combining data from both strands can improve confidence. For this reason, we wrote the muSeq assembly pipeline to work with data from both strands when available.

We designed the muSeq program to assemble the two highly similar templates present in a diploid genome. These methods, however, generalize to genomes of any ploidy or to mixtures of genomes. As a demonstration, we combined read data from both strands and all four family members, generating a mixed *in silico* sample that has four haplotypes over the region. Using this mixed data as input for the muSeq algorithm, we correctly assemble all four distinct haplotypes (A–D). Upon examination, we find that every mutated template is constructed from reads derived from only one of the eight input libraries. This result also suggests that the algorithm does not generate chimeric assemblies, an observation that we use to explore parameter settings (see Supplementary Methods, informatics step 1).

After the assembly of mutated templates, we confirm the validity of a template by mapping the mutated reads back to the assembly. We noticed that all templates that assemble to full-length have a median coverage of 100× or greater. Since many templates are covered to depths of 1000x, we decided to test the performance of our assembly algorithm when presented with lower coverage data.

We began with a single library (top strand, mother) containing 1.8 million 300-bp reads over a 5.1 kb region for coverage >100 000 reads per base position. As in Supplementary Figure S1, we estimate the number of templates by counting distinct mutated *k*-mers for each position in the final assembly. Using a minimum *k*-mer count of 20, we estimate 200 template molecules for an average of ∼530× coverage per position per template. We therefore down-sample the reads from the sequence library so that the coverage per-template ranges across five orders of magnitude: from 512x per template down to 16× per template (Figure 2D). We find that at the highest coverage, nearly all templates are recovered at full length (150 of 200). There is a marginal reduction at 256× coverage, and at 128× average per-template coverage, we still recover ∼100 full-length templates, sufficient for a correct and confident haplotype assembly.

### Region 2: the HLA-B locus

We next applied muSeq *de novo* assembly to a highly polymorphic region: a 4.8 kb genomic fragment on chromosome 6 that contains the HLA-B gene. This region is notoriously variable in the human population, but due to its medical importance, many haplotype sequences have been assembled and archived in the IMGT/HLA genomic sequence database. Using just the single canonical reference sequence for mapping whole genome reads often results in poor and uneven coverage. In the family chosen for this study, the coverage is sufficient to guess that there are four different (unresolved) alleles in the parental genomes, with all four alleles appearing in the children. For this region, we assembled each family member using top-strand data only. The muSeq protocol (Figure 3B) generates two assemblies per person, eight in total. These assemblies occurred in matched pairs of four distinct sequences, two in each parent, and two in each child, consistent with inferences about transmission made from the WGS data. In Figure 3, we mark up the variants relative to the canonical reference sequence. There are far more variants in this locus than in the previous locus, and more deletions and insertions. We queried the IMGT/HLA genomic sequence database, and found a perfect match to an HLA-B variant for each of the four haplotypes. As we did for the previous locus, we mixed the reads from all the libraries, thereby creating a tetraploid assembly problem. MuSeq returns the same four haplotypes.

### Region 3: enzymatic mutagenesis

We successfully assemble and haplotype 5 kb templates using partial bisulfite conversion as the means of mutagenesis. Unfortunately, the bisulfite protocol degrades templates, and performing muSeq on much longer templates results in unacceptable losses of efficiency. Soon after our first successes with 5 kb assembly, a commercial kit became available that deaminates unmethylated cytosine enzymatically. The enzyme is processive, and so cannot be used to imprint a random pattern of **C** to **U** conversions. But by incorporating mixtures of dCTP and 5-methyl-dCTP into the first copies of templates, we hoped to create nearly random patterns of **C** to **U** conversion in copies of a given template at any desired proportion.

We generated three converted sequence libraries for the mother: one top-strand library and two bottom-strand libraries, from the 10.5 kb Region 3 (chr18:11 666 735–11 677 605). We then tested the libraries from the new protocol in our established assembly pipeline using the same assembly parameters as we established for the 5 kb region. We assembled each library separately, but also assembled an *in silico* mixture of the two bottom-strand libraries. As shown in Figure 3C, the algorithm assembled both haplotypes consistently. When compared with the family transmission data generated by whole-genome sequencing, we confirm that the assemblies are all without error (see Supplementary Table S2 for all haplo-assemblies and inferred WGS haplotypes).

Finally, we examine the template assemblies from the *in silico* mixture of the two bottom-strand libraries. As in Region 1, we count the number of distinct mutation patterns in the mutated libraries for each unmutated *k*-mer (Supplementary Figure S2) and estimate ∼600 full-length original templates. The extended contigs generated in the first step of the pipeline include 104 contigs greater than 10 kb in length and 645 contigs greater than 5 kb in length (Supplementary Figure S3). If some of the assemblies have errors in joining the extended contigs, half of those errors would cross between the two libraries. Since the libraries are mixed *in silico*, we can determine in retrospect whether a contig is built from reads from one or both libraries. From the 2234 extended contigs >1.5 kb in length, we identified two that exhibited chimerism, for an estimated rate of 0.2%. On examination, the two chimeric assemblies were short (4 kb and 3.5 kb), and the chimeric junctions occurred in regions of low mutational complexity.

Another possible source of chimerism is recombination between templates during PCR. We examined the rate of strand-recombination within one bottom-strand library by looking for templates that are inconsistent for haplotype across their length (see Supplementary Figure S4). From 1313 extended contigs greater than 1.5 kb in length, only one template shows evidence of strand recombination. This approach can be used to monitor for chimeric recombination which may depend on PCR conditions.

Lastly, we examined the rate of PCR error in template assemblies (see Supplementary Methods). Over all assigned templates, we estimate the rate of SNV error to be ∼7.4 × 10^–4^ and the rate of indel error to be ∼7.7 × 10^–5^. Upon further examination, 68% of indel errors occur at mononucleotide or dinucleotide microsatellite sequences and 92% occur at positions which are mononucleotide repeats under full genomic conversion. Even at these positions, the error rate is sufficiently low that aggregation results in the correct haplotype sequence.

## DISCUSSION

Short reads are excellent for measuring local genomic variation over most of the genome. When short reads cannot be assembled unambiguously, for example, over repetitive regions or when phasing haplotypes over regions of low variation density, then long reads are needed. Unfortunately, while short reads are virtually a commodity, long read platforms require expensive dedicated equipment. Moreover, despite industrial-scale investment over many years, long-read sequencing platforms remain costly and have high error rates. We proposed a solution using short-reads for highly accurate long-range assembly: imprint each template molecule with a unique mutational signature (5). To realize this solution, we developed muSeq, a method for embedding random mutation patterns using partial bisulfite conversion of **C** to **U** (6). We showed that with muSeq we could do assembly, count initial templates, and further improve sequence accuracy.

The first version of muSeq faced two major limitations. First, partial bisulfite conversion is destructive, limiting the length of templates we can assay. Second, our informatics relied on a reference genome, restricting applications to well-characterized genomic regions. The present study updates muSeq with a gentler protocol for template mutagenesis and new algorithms for assembly that do not require a reference genome. These improvements enable short-read sequencing platforms to perform highly accurate long-range assembly in virtually any context.

In the past and present study, we used the sodium bisulfite reaction for partial mutagenesis of templates. Although the bisulfite reaction is harsh, after controlling time, temperature, and reagent proportion (see Supplementary Methods), we can routinely recover mutated templates up to 5 kb. However, obtaining significantly longer templates with this protocol was problematic. To overcome this constraint, we devised a gentler procedure using enzymatic deamination of cytosine. In our initial attempts, limiting enzymatic activity did not achieve random conversion. Therefore, we first incorporate methyl-cytosine randomly into copies of the template. We then subject the templates to the TET2 enzyme, which oxidizes the methyl-cytosines. In the next step, we add the APOBEC enzyme which converts the non-oxidized cytosines to uracil. The result is a unique random pattern written into each copy of the initial template. (We note one important detail in this protocol. We could not replicate long templates following TET2 and APOBEC treatment using a standard mixture of dNTP. However, we achieved efficient amplification of the mutagenized templates if we included methyl-dCTP in the mixture.) We demonstrate this protocol on 10 kb templates; and based on the high yield of mutagenized full-length templates, we project the method to be limited only by the length of molecules that can be replicated *in vitro*.

The first version of muSeq borrowed from existing methods in bisulfite methylome sequencing to align sequence reads and determine mutation patterns. These methods require a reference genome. In contrast, for *de novo* assembly, we use only the existing sequence variation in the reads to assemble the mutated template molecules. Our template assembly method combines elements of graph-based assembly (de Bruijn graphs) and fast-mapping alignment (suffix-array read-mapper) to build and join consensus assemblies. Though crude, our method recovers in full-length between 50 and 90% of initial template molecules from amidst hundreds of nearly identical sequences. There are likely other approaches for template assembly that would improve on our results and we discuss some suggestions below.

In the previous version of muSeq, we used the reference genome to restore mutated positions to their original base. For *de novo* assembly, we devised a new approach that uses a set of unmutated sequence reads over the same region to restore mutated positions. The approach is analogous to building a set of very short reference genomes from the *k*-mers in the unmutated reads, weighted by their frequency. This approach unmasks 99% of mutated positions, leaving the remaining 1% which are **C**/**T** heterozygous positions and imperfect repeats. These positions cannot be resolved without additional information because either base is compatible with the unmutated data. To resolve such positions with confidence requires aggregation over identical templates with different mutation patterns.

This is precisely what we have when sampling a region of the human genome with the important caveat that the input genomes are *diploid*, and so we have *two* sets of identical templates, one set for each haplotype. What makes this problem vexing is that the two haplotypes are often very similar to each other, but not identical, with distinct differences of about 1 SNV per kb. Since we target regions of 5 kb and greater, we can use these SNVs to phase the haplotypes. For this paper, we built a maximum likelihood method to identify the best two haplotypes to explain the observed SNV data. Our method phases **C**/**T** heterozygous positions, handles data from both strands, and scales up to any ploidy.

Having split the haplotypes by SNVs, we conclude by aggregating contigs from the same haplotype to recover the full haplotype sequence. Aggregation returns the most likely haplotype sequence, averaging over template differences that arise from somatic variation or polymerase error. These differences are typically sparse, and for most positions in the sequence, better than 99% of templates will agree on the base. The aggregation step also generates a confidence score for each base in the assembly, reflecting the coverage and error at that position. Positions in the assembly that are still masked present a special case: either all of the templates will agree on **T** (the original base was **T**) or half the templates report **T** and the other half a **C** (the original base was a **C**). For these positions, we require higher coverage to obtain the same confidence in the base. Including data from the opposite strand, in which the complementary base is not subject to mutation, will also improve the confidence of the base call.

We chose to develop our protocols and informatics and demonstrate the validity of muSeq in solving a very difficult genomics problem, namely haplotype assembly. Because we have samples from a family pedigree, we could verify our results by observing expected patterns of transmission from parents to children. We also had whole-genome sequence data for the whole family, which confirmed haplotype phasing for well-mapped genomic regions. Haplotype assembly presents a useful test for muSeq since correct phasing requires assembling strands that are otherwise nearly identical. We chose three regions to test our methods. The first region we selected for its low rate of variation, making phasing impossible from short reads alone. The second region we selected for its high rate of variation: the HLA locus is notoriously variable in the population, making reference mapping difficult. Further, because of its importance in predicting graft-host response, having an accurate read of these loci is of practical value. Our third region was well-mapped by the whole genome data, but otherwise selected at random for its greater length.

The muSeq method recovered the correct haplotype sequences for all three regions. Further, we obtained full-length recovery of nearly every mutated template, even faced with the presence of hundreds of similar templates. We stressed the ability to accurately assemble templates by *in silico* mixing of different individuals from the same family. We find that nearly all templates identified in the family mixture derive their reads from one of the eight sequencing libraries. This strongly suggests that the assembly algorithms rarely make errors in assembly.

Sequence assembly is a read-intensive process, typically requiring read coverage between 100 and 200× (9–11). The de novo muSeq assembly pipeline requires about that level of coverage for each mutated template. If there are 100 mutated templates in the mix, then muSeq requires ∼10 000× coverage to assemble them all. From our down-sampling experiments, we estimate that nearly all templates with better than 100x coverage are successfully assembled to full-length.

To develop muSeq into a universal long-read sequencing technology requires only a few additional improvements. To apply to a generic sample, we would not use targeted sequence primers, but instead, ligate mutation-resistant primers onto the input molecules. These primers would include either methyl-C or use a reduced alphabet that omits C, to evade conversion during mutagenesis. These primers could then be used for post-conversion amplification.

Our assembly pipeline successfully demonstrates that all the sequence information is present for accurate template assemblies, and that assembly does not require a preexisting reference genome. However, while functional, this assembly pipeline is likely not final. One recurrent problem in template assembly is early-round polymerase errors in copies of the mutated templates. When these errors introduce single-nucleotide errors, they can be corrected by properly tuning the disruption parameter. But insertion-deletion errors, which are common in some sequence contexts, such as direct short repeats, are not well-handled by the present pipeline. We address this problem separately in another manuscript (12), where we demonstrate that partial mutagenesis can accurately recover microsatellite repeat lengths with applications beyond genome assembly.

MuSeq is, in effect, a protocol to use accurate short-read sequencing platforms to obtain accurate long single template sequences. We illustrated its operation on many templates, all from specific loci and all with the same registry. But there is no fundamental obstacle to its application on more complex populations of molecules, which may arise from multiple loci, even from multiple complex cells in a sample, for example, as one might encounter in a cancer biopsy. Our approach bears some similarity to the Linked-Read method of the 10X Genomics Chromium platform (13). However, Linked-Reads have sparse information over each template molecule and expressly do not assemble the templates, so it cannot resolve complex or repetitive regions. Moreover, Linked Reads rely on reference genome mapping to co-localize variants for genomic phasing and are of limited utility in de novo assembly (13). MuSeq makes the Illumina platform comparable to expressly long-read sequencing platforms, probably at similar costs, but with greater accessibility and higher accuracy.

## DATA AVAILABILITY

Sequencing data is available in the European Genome-Phenome Archive (EGA) with Study ID: EGAS00001005899 and dataset ID: EGAD00001008444.

Source code is available at https://github.com/levycshl/museq/.

## Supplementary Material

gkac592_Supplemental_Files
