# Supplementary material for: Targeted de novo phasing and long-range assembly by template mutagenesis
Source: Nucleic Acids Res. 2022 Jul 13;50(18):e103. doi: 10.1093/nar/gkac592 (PMC9561374; doi:10.1093/nar/gkac592)
Supplement: gkac592_Supplemental_Files [file gkac592_supplemental_files.zip › muSeq2022.REV2_Supplementary Figures and Tables.pdf]

Supplementary Figures and Tables

Supplementary Figure 1

Using the reference sequence over region 1, we list all 5100 *k*-mers in order. Using the unmutated data, we count how many times each *k*-mer was observed in the reads. This is shown in black and matches the y-axis on the right. The coverage is about 50,000x and the dips in coverage correspond to positions where the family differs from the reference. Using the mutated data, we assign a mutated *k*-mer to a position if its count in the mutated data is greater than 20 and it is compatible with C->T mutation or G->A mutation from the reference *k*-mer at this position. This count of mutated coverage is shown in blue. If at each position, we collapse mutated *k*-mers that differ by only a single base, we obtain the rollup count shown in orange. Both the blue and orange curves are similar and we estimate ~725 mutated templates.

Supplementary Figure 2

As in Supplementary Figure 1, we use the reference genome this time over region 3, listing all 10,800 *k*-mers in order. Here, the mutated data are restricted to the two bottom-strand libraries and we estimate ~600 templates in total.

Supplementary Figure 3

The distribution of initial and extended contigs for region 3, the *in silico* mixture of the two bottom-strand libraries. Our assembly yields 100 full-length templates where we expect 600 templates in total (see Supplementary Figure 2).

Supplementary Figure 4

Each point shows the log-likelihood that a contig matches either haplotype 1 (x-axis) or haplotype 2 (y-axis), for contigs from one bottom strand library of Region 3. Contigs are colored blue if they are assigned to haplotype 1, orange if assigned to haplotype 2, and green if they are unassigned. About 80% of contigs are assigned to one of the two haplotypes, and those that are not are typically short. There is one exception, a contig with seven variants (marked with the yellow arrow); the first four are from one haplotype and the last three from the other. This is consistent with a low rate of template recombination during PCR.

Supplementary Table 1

We complete a full run of the muSeq assembly pipeline for region 1. We compute haplotype assemblies individually for mother, father, proband and sibling (“mo”, “fa”, “pr”, and “si”) or from the pool of all four individuals (“all”). In all five cases, we use data either from the original top (“ot”), the original bottom (“ob”) or pool data from both strands (“both”). Each of the individual assemblies generates two haplotypes (“h1” and “h2”) and the family data generates four haplotypes (“h1”, “h2”, “h3”, and “h4”). We compare the haplotype data to the reference genome on the interval chr14:92,426,501-92,432,500 assigned in the table to positions 0-5999.

We genotype the family from whole-genome sequence data (columns "mo", "fa", "pr" and "si") and apply a parsimonious algorithm to determine the parental haplotypes for each SNP from the perspective of the proband: mother/father allele transmitted/not transmitted to proband ("MT_pr", "MNT_pr", "FT_pr", "FNT_pr") and likewise for the sibling. From this data, we conclude that the children agree for the maternal allele over this region and diverge for the paternal allele.

We align each assembled haplotype ("who_strand_haplotype") against the reference genome and record both the aligned base ("base_who_strand_haplotype") and the quality score of that position ("qual_who_strand_haplotype"). Quality scores are reported in the Phred scale, $-10*\log_{10}$(prob of error). For the most part, the assemblies are in perfect agreement with expectation and the few errors that are observed occur with low quality (<7 or 80% confidence) and at just a few positions (i.e. pos = 5245, 5276). In contrast, the vast majority of bases (98%) report the maximum quality score of 91, i.e. confident up to an error of 1 in a billion.

Supplementary Table 2

We compute a full run of the muSeq assembly pipeline for region 3. We have three separate libraries from the mother's DNA: two libraries ("lib1" and "lib2") from the original bottom ("ob") and one library from the original top ("ot"). We also run the two original bottom libraries together ("both"). As in Supplementary Table 1, we list two haplotypes as "h1" and "h2" for each assembly. We compare the haplotype data to the reference genome on the interval chr18:11666701-11677600 assigned in the table to positions 0-10899, otherwise, as in Supplementary Table 1.

Supplementary Figure 1

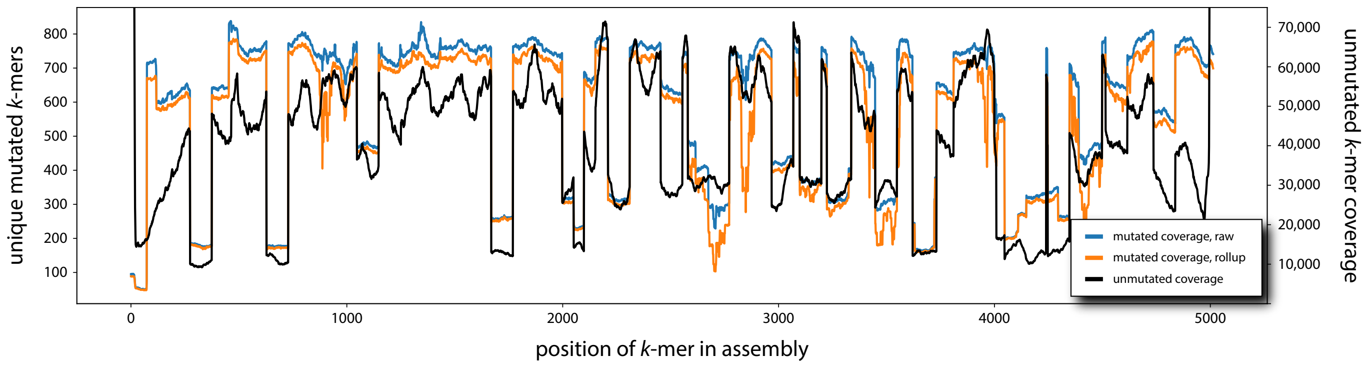

Supplementary Figure 1: Using the reference sequence over region 1, we list all 5100 k-mers in order. Using the unmutated data, we count how many times each k-mer was observed in the reads. This is shown in black and matches the y-axis on the right. The coverage is about 50,000x and the dips in coverage correspond to positions where the family differs from the reference. Using the mutated data, we assign a mutated k-mer to a position if its count in the mutated data is greater than 20 and it is compatible with C->T mutation or G->A mutation from the reference k-mer at this position. This count of mutated coverage is shown in blue. If at each position, we collapse mutated k-mers that differ by only a single base, we obtain the rollup count shown in orange. Both the blue and orange curves are similar and we estimate ~725 mutated templates.

Supplementary Figure 2

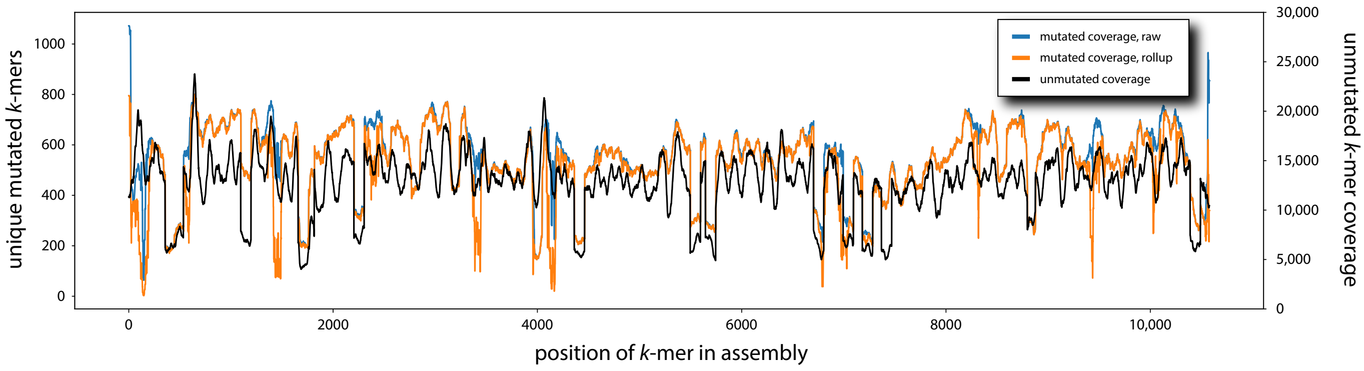

Supplementary Figure 2: As in Supplementary Figure 1, we use the reference genome this time over region 3, listing all 10,800 k-mers in order. Here, the mutated data are restricted to the two bottom-strand libraries and we estimate ~600 templates in total.

Supplementary Figure 3

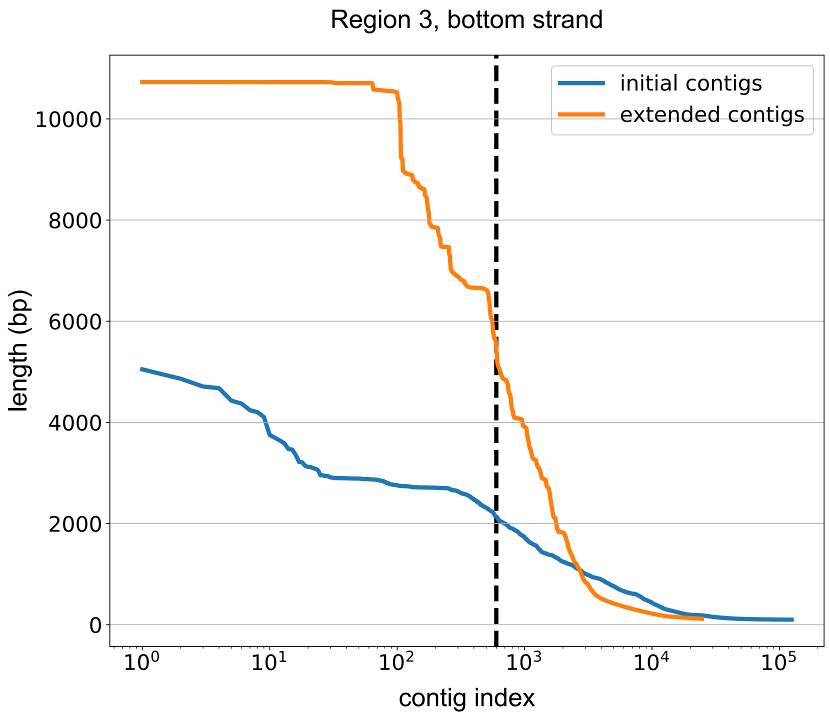

Supplementary Figure 3: The distribution of initial and extended contigs for region 3, the in silico mixture of the two bottom-strand libraries. Our assembly yields 100 full-length tem-plates where we expect 600 templates in total (see Supplementary Figure 2).

Supplementary Figure 4

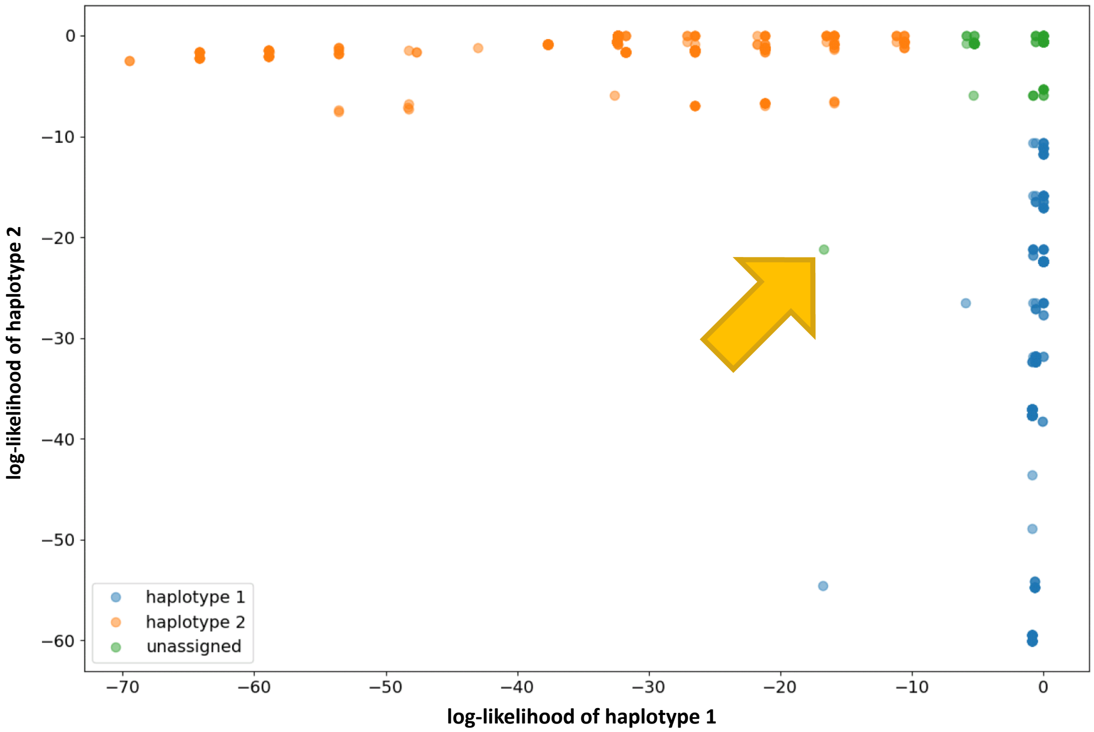

Supplementary Figure 4: Each point shows the log-likelihood that a contig matches either haplotype 1 (x-axis) or haplotype 2 (y-axis), for contigs from one bottom strand library of Region 3. Contigs are colored blue if they are assigned to haplotype 1, orange if assigned to haplotype 2, and green if they are unassigned. About 80% of contigs are assigned to one of the two haplotypes, and those that are not are typically short. There is one exception, a contig with seven variants (marked with the yellow arrow); the first four are from one haplotype and the last three from the other. This is consistent with a low rate of template recombination during PCR.
