# Supplementary material for: Targeted de novo phasing and long-range assembly by template mutagenesis
Source: Nucleic Acids Res. 2022 Jul 13;50(18):e103. doi: 10.1093/nar/gkac592 (PMC9561374; doi:10.1093/nar/gkac592)
Supplement: gkac592_Supplemental_Files [file gkac592_supplemental_files.zip › muSeq2022.REV2_Supplementary Methods.pdf]

Supplementary Methods

Sample

The cell sources SSC12592, SSC12594, SSC12596, and SSC12597 are EBV immortalized lymphocyte cultures belonging to the Simons Simplex Collection (SSC). These cells were obtained from the RUCDR Infinite Biologics (formerly Rutgers University Cell and DNA Repository). The SSC is a core project of the Simons Foundation Autism Research Initiative (SFARI).

The SSC cells were cultured in T25 flasks using tissue culture media consisting of RPMI, 15% FBS, 1× Pen Strep, 1× MEM NEAA, and 1× GlutaMAX. Cells grew in suspension and could form aggregates. When the confluency reached approximately 1 million cells per ml, the cells were passaged. During the passage, the cells were pipetted gently to break up clumps. The cells were counted and then reseeded at a density of about 100,000 per ml. Cells were passaged every 3-4 days.

During cell collection, the desired number of cells were spun down at 1000 rpm in a 15 ml conical tube. The media was aspirated off, and the cells were resuspended in 500 µl cold 1× PBS and transferred into a 1.5 ml tube. The tube was spun at 1000 rpm again, and the PBS was aspirated off. The entire collection process was done on ice and in refrigerated centrifuges.

The genomic DNA was extracted from the cell pellets using DNeasy Blood & Tissue Kit (Qiagen 69504) and quantified using NanoDrop 2000 (Thermo Fisher Scientific).

Nested PCR design

We utilized partial C-to-U mutation to label each long single-stranded DNA template with a unique mutation pattern. These partially mutated long templates were then amplified, sonicated, sequenced, and assembled. In this study, we introduce two bench methods to perform partial C-to-U mutation and amplification. These two methods, named partial bisulfite mutation and APOBAC-based enzymatic conversion, will be described in the following two sections. These two methods are different in chemistry, but share similar nested PCR workflows.

The nested PCR workflow included two sequential PCRs. The first PCR covered a larger genomic region than the second PCR product. We performed partial C to T conversions on the first PCR product using one of the two mutation methods, and then performed the second PCR to amplify the target region. The second PCR product was sonicated and sequenced using Illumina short-read sequencers. The nested PCR approach increased both the specificity and yield of the product.

After the first PCR amplification, we used the Bioanalyzer machine to measure the concentration of the target molecules. This enabled us to control the expected number of molecules for the next processing step (bisulfite or enzymatic conversion). When performing the bench protocols for the first time, we used a serial dilutions of the 1st PCR post-conversion products as inputs to the 2nd PCR step. This allowed us to identify concentrations that yield sufficient material for library preparation.

The second PCR primers were designed prior to the first PCR primers. To design the second PCR primers, we checked the DNA sequences both upstream and downstream of the target region on the same strand. We looked for 18-25 nucleotide G-rich (or C-rich) sequences at both sides of the target region from the same strand. These sequences served as the candidates for second PCR primers. Multiple G-rich or C-rich sequences were easy to find within 500 bp upstream and downstream of the target regions. The second PCR was strand-specific, meaning the second PCR primers would amplify one of the two strands much more efficiently than the other. The successfully amplified strand had two G-rich sequences as PCR primer binding sites located upstream and downstream of the target region. The other strand had two C-

rich sequences, which were partially mutated, at the corresponding primer binding sites. As a result, the primer binding and amplification were not efficient.

The quality of PCR primers was examined using the OligoAnalyzer tool from IDT to ensure similar melting temperatures and avoid primer hetero-dimers. After designing the PCR primers for the second PCR, we designed the first PCR primers to amplify a larger region covering the second PCR product. The first PCR primers do not have G/C restrictions and were designed with the aid of "Primer3" online primer designing software (https://bioinfo.ut.ee/primer3-0.4.0/).

Method one: Partial bisulfite mutation

A modified bisulfite sequencing protocol was used to randomly convert about 50% of the cytosine to uracil. The partial bisulfite conversion was applied to the first PCR product. In the first PCR, 25 cycles of amplification were performed using LA Taq DNA Polymerase Hot-Start Version (Takara Bio USA, RR042A), with about 100 ng genomic DNA as the starting material (see PCR recipes and conditions below). The length and quantity of the PCR product were measured by Agilent 2100 Bioanalyzer.

Components	Volume (unit: ul)
10× LA PCR Buffer (Mg^{2+} plus)	5
dNTP (10 mM each)	2
Forward PCR primer (10 µM)	2
Reverse PCR primer (10 µM)	2
Isolated genomic DNA (50 – 100 ng/µl)	1
TakaRa LA Taq HS	0.5
H_2O	37.5
	**Total volume**: 50

PCR Conditions for the 1st PCR	
94 °C	2 minutes
**25 cycles of:**	
98 °C	10 seconds
Annealing Temperature	30 seconds
68 °C	2 minutes per kb
**Then:**	
72 °C	10 minutes
4 °C	infinite

We added 200 ng of salmon sperm DNA (Invitrogen 15632011) as carrier DNA into each bisulfite reaction. We performed bisulfite treatment of the first PCR product following the manufacturer's protocol

of the EZ DNA Methylation-Direct kit (Zymo Research, D5020) with the following modifications. First, 130 ul of CT conversion reagent was added after, instead of before, the 98 °C denaturation step. Second, the time and temperature of bisulfite treatment were 50 minutes and 55 °C to achieve a C to T conversion rate of about 50%.

DNA polymerase Phusion U Hot Start DNA Polymerase (F555, Thermo Fisher Scientific) is used to amplify bisulfite-treated templates following the recipe and conditions below.

Components	Volume (unit: ul)
5× Phusion GC Buffer	10
dNTP (10 mM each)	1.2
Forward PCR primer (10 µM)	1.9
Reverse PCR primer (10 µM)	2
Isolated genomic DNA (50 – 100 ng/µl)	1
Bisulfite-treated DNA (diluted)	1
Phusion U HS DNA polymerase	0.6
H_2O	32.3
	**Total volume**: 50

PCR Conditions for the 2nd PCR	
98 °C	30 seconds
**5 cycles of:**	
98 °C	15 seconds
Annealing Temperature minus 2°C	45 seconds
72 °C	0.8 minutes per kb
**33 cycles of:**	
98 °C	10 seconds
Annealing Temperature	30 seconds
72 °C	0.8 minutes per kb
**Then:**	
72 °C	5 minutes
4 °C	infinite

Method two: APOBAC-based enzymatic conversion

This approach is an alternative to the bisulfite approach to achieve a 50% C to U conversion for each template. However, the enzyme-based method is considered a more gentle method than bisulfite-based C-to-U conversion, so it works for an even longer region. We successfully demonstrated in the paper that this approach worked for a region longer than 10 KB.

We still used nested PCR workflow as in Method one, but here we incorporated a mixture of standard dCTP and 5-methyl-dCTP (NEB N0356S) into the PCR product using the following PCR recipe so that each PCR product has a random methyl-C pattern.

Components	Volume (unit: ul)
5× Phusion GC Buffer	10
dATP (10 mM)	1
dTTP (10 mM)	1
dGTP (10 mM)	1
dCTP (10 mM)	1
5-methyl-dCTP (10 mM)	0.5
Forward PCR primer (10 µM)	2
Reverse PCR primer (10 µM)	2
Isolated genomic DNA (50 – 100 ng/µl)	1
Phusion U HS DNA polymerase	0.5
H_2O	30
	**Total volume**: 50

**PCR Conditions for the 1st PCR**	
98 °C	1 minute
**26 cycles of:**	
98 °C	15 seconds
Annealing Temperature	45 seconds
68 °C	1.5 minutes per kb
**Then:**	
68 °C	10 minutes
4 °C	infinite

We used Enzymatic Methyl-seq Conversion Module (NEB, E7125S) to convert standard cytosine in the PCR template to uracil, which left 5-methylcytosine (5mC) unchanged. It contained two steps. The first step used the TET2 enzyme to oxidize 5-methylcytosines, so the methylcytosines would be protected from being converted by APOBEC. The second step used the APOBEC enzyme to convert the non-oxidized cytosines to uracils. APOBEC itself has a heavily biased C to T conversion rate based on sequence context, so using APOBEC alone without the involvement of 5-methyl-dCTP and TET2 enzyme would not achieve a random mutation.

We followed the manufacturer's protocol except for the following modifications. First, before denaturation of DNA, we added 1 ul of 100 ng/µl of salmon sperm DNA to the TET2-treated DNA product as carrier DNA. Second, the time and temperature for APOBEC treatment was 20 minutes and 37 °C. We used Q5U Hot Start High-Fidelity DNA Polymerase (NEB M0515) in the second PCR. The recipe and condition was as follows.

Components	Volume (unit: ul)
5× Q5U Reaction Buffer	10
dATP (10 mM)	1
dTTP (10 mM)	1
dGTP (10 mM)	1
dCTP (10 mM)	0.75
5-methyl-dCTP (10 mM)	0.75
Forward PCR primer (10 µM)	2
Reverse PCR primer (10 µM)	2
mutated DNA (diluted)	1
Q5U DNA Polymerase	0.5
H_2O	30
	**Total volume** : 50

**PCR Conditions for the 2nd PCR**	
98 °C	50 seconds
**35 cycles of:**	
98 °C	10 seconds
Annealing Temperature	30 seconds
68 °C	1.5 minutes per kb
**Then:**	
68 °C	10 minutes
4 °C	infinite

Sequencing library generation

The second PCR was to amplify the mutated single-stranded DNA templates, and the product was checked by Agilent Bioanalyzer. For long products, if necessary, we performed a size-selection step to only select the DNA of the right size. Size selection was performed using the BluePippin™ (Sage Science) to enrich the targeted fragment and eliminate the shorter off-target fragments. Samples were run on the 0.75% agarose cassette with the S1 external marker. Samples were then purified with 1.8x AMPure XP beads and analyzed with a High Sensitivity chip on the Agilent Bioanalyzer. The nested PCR product was sonicated by Covaris E220 Focused-ultrasonicator to an average fragment length of 400 bp. The sonicated product was repaired using NEBNext Ultra II End Repair/dA-Tailing Module (NEB, E7546) followed by NEBNext Ultra II Ligation Module (NEB, E7595). We ligated Illumina sequencing adapters with customized indexes. A final 8-cycles of PCR using Illumina sequencing adapters (P5 and P7) and default sequencing primers was performed to generate the final sequencing library. The library was quantified using Agilent Bioanalyzer and sequenced in an Illumina MiSeq sequencer.

Primary informatic pipeline

The informatics pipeline is divided into nine computational steps. These steps are the atomic elements of the muSeq pipeline and are detailed here in the order they appear in the standard implementation. The main muSeq program takes five inputs. The first parameter specifies the location of the configuration file, **config_filename**. The configuration file contains all the parameters needed for running muSeq. In the code, parameters are typically written in **ALL_CAPS** and within this document, we will also write them in bold. Where appropriate, we will also include the default value. The next two command line parameters are the input directories, **BS_0_raw_directory** and **BS_1_raw_directory**. BS_0 contains the unmutated sequence reads and BS_1 contains the mutated sequence reads, and each directory contains two files, “r1.fastq.gz” and “r2.fastq.gz” corresponding to the gzipped paired-end reads. The last two inputs are a **parent_data_directory** and a **short_name,** which together name the target directory for our output.

Step 1: Unpack the reads

In the first step, we make the needed target directories and then unzip the reads into them. At this time, we also replace the original read names with integers specifying their order in the file. With this convention, the read name identifies the location of the read in the file.

Step 2: Counting *k*-mers

In the second step, we run the jellyfish program over the reads for both mutated and unmutated data. This step loads parameters from the configuration file that include the size of the kmer (**KMER_SIZE**), the smallest kmer count we are willing to consider (**MIN_MER_COUNT** = 2). Other parameters specify the path to the jellyfish program (**JELLYFISH**), the amount of memory to allow (**JELLYFISH_MEM** = 100M) and the number of threads (**JELLYFISH_THREADS** = 10). This step also takes the jellyfish “mer_count.jf” file and outputs a human readable “kmer_list.txt”.

Step 3: Build initial contigs

Selecting all mutated kmers (BS1) with counts greater or equal to **MIN_DB_COVER** (= 10), we build the de Bruijn graph. Briefly, the selected kmers and their complements are loaded into a dictionary with counts. We initialize the set of “done” kmers to an empty set. We then start with any kmer that is not in the done set and build the longest unambiguous path forward and backward from that node, joining them

to comprise a single ***initial contig***. Each kmer in that contig is added to the "done" set and we repeat until all kmers in the de Bruijn graph are "done." The result is a set of initial contigs stored in fasta format in the file "contigs.fa". The contigs are ordered by length and named according to their index in the file.

In addition to the de Bruijn graph, we also record the *k*-mer count from the **first** and **last** *k*-mer of each contig. A **spur** is a contig arising from low-frequency errors near the ends of reads. Therefore, it should have three properties: First, since the error occurs in the last *k* bases of the read, the length of a spur contig cannot exceed 2*k* - 1. Second, a spur can have only a single neighbor in the de Bruijn graph, the source sequence where the error arose. And third, since it arose by error, the coverage of the spur should be significantly less than the alternative, as determined by the parameter **SPUR_TO_ALT_MAX_RATIO** (0.2).

To find spurs, we begin with the set of all contigs shorter than 2*k* -1 with exactly one link to the head or tail of another contig. The neighboring *k*-mer, we refer to as the "source." We then iterate over its neighbors (excluding the spur) and record its best-covered neighbor as the "alternative." If the coverage of the spur is less than **SPUR_TO_ALT_MAX_RATIO** * (coverage of alternative), we remove the spur from the de Bruijn graph; otherwise, it remains. After removing spurs, we simplify the de Bruijn graph as needed and report a list of "spurless" contigs.

Step 4: Map mutated reads to initial contigs

We use the mumdex mapper (**MUMDEX_FASTQ**) to map mutated reads to the initial contigs. These maps comprise the set of longest possible unique exact matches between the reads and the initial contigs. These are commonly referred to as "**m**aximal **u**nique **m**atches" or **MUM**s. We find these maps using the suffix array implementation from the mumdex package (https://github.com/docpaa/mumdex). A second configuration parameter **MIN_MUM_LENGTH** (40) specifies the shortest match length the mumdex program will return. The output is in the form of a text file, "contig_maps.txt". This file has a single row for each match and seven values per match:

1. The name of the read (integer)
2. Read 1 or read 2 (0 or 1)
3. Contig name (integer)
4. Contig position (integer)
5. Read position (integer)
6. Match length (integer)
7. Reverse complement to match (-) or not (+)

Step 5: Annotate mumdex maps

The mumdex maps provide information about exact match but do not specify the quality of a map outside beyond the match. Therefore, for each mumdex map from step 4, we align the read to the contig according to the MUM and add four additional pieces of information to each map:

8. Read-to-contig intercept ($\mathbf{S_{RC}}$, integer)
9. Read-to-contig slope ($\mathbf{F_{RC}}$, +1/-1)
10. Hamming distance for read/contig alignment (**match**, integer)
11. Length of overlap in read/contig alignment (**total**, integer)

The read-to-contig slope and intercept treat an alignment as map between coordinate frames, converting a position in the read (x_R) to a coordinate position in the contig (x_C) such that:

$$\mathbf{x_C = S_{RC} + F_{RC} * x_R}$$

The convention enables computational manipulations like inverting maps (from contig-to-read) and composing maps (A-to-B) and (B-to-C) yield (A-to-C):

($\mathbf{S_{BA}}$, $\mathbf{F_{BA}}$) = (-$\mathbf{F_{AB}}$*$\mathbf{S_{AB}}$, $\mathbf{F_{AB}}$) and

($\mathbf{S_{AC}}$, $\mathbf{F_{AC}}$) = ($\mathbf{S_{BC}}$ + $\mathbf{F_{BC}}$*$\mathbf{S_{AB}}$, $\mathbf{F_{AB}}$*$\mathbf{F_{BC}}$)

This is particularly useful because the edges of a de Bruijn graph impose a set of coordinate-change relationships between contigs. By composing coordinate maps, we can convert a read alignment from one contig to a read-alignment in a neighboring contig. This is important in the following step.

Step 6: Extend contigs with annotated maps

While atomic in the scope of the pipeline, the contig extension step involves several interacting sub-components. The main modules are (1) identifying high-quality maps from the annotated maps, (2) determining map extensions along the de Bruijn graph, (3) for each contig, aligning all well-mapped read-pairs using map extensions as needed, (4) for each contig, extending where the consensus is well-covered and unambiguous, and (5) identifying compatible extensions and joining them into **final contigs**.

In the first step, we identify two sets of maps:

1. ***Good-ratio maps*** are those with at least **MIN_MAP_RATIO** (0.985) proportion of bases in agreement between the read and the contig.
2. ***Top-quality maps*** are good-ratio maps that also have at least **MIN_MAP_LENGTH** (120) base pairs of overlap between the read and the contig.

We are interested in mapping read-pairs to contigs. For these purposes, we consider a read-pair as **well-mapped** to a contig if at least one read in the pair has a top-quality map to the contig.

Since we consider only well-mapped read pairs, we will only extend contigs that could have a top-quality map and therefore have a minimum length of **MIN_MAP_LENGTH**. For each sufficiently long contig, we travel along the de Bruijn graph **EXTENT** (1000) bp forward from the tail and backwards from the head using a breadth-first search. Given two contigs **A** and **B** such that **A** and **B** share an edge in the de Bruijn graph, the edge joins either the head or tail of the **A** contig to the head or tail of the **B** contig, for four possible configurations. If two contigs share an edge in the K-mer de Bruijn graph, they must have a K-1-mer in common. We will denote this **K-1**-mer as **X**. The position and orientation of **X** in each contig determine the coordinate maps ($\mathbf{S_{AX}}$, $\mathbf{F_{AX}}$) and ($\mathbf{S_{BX}}$, $\mathbf{F_{BX}}$). For contig A (and similarly B)

1. If head and forward: ($\mathbf{S_{AX}}$, $\mathbf{F_{AX}}$) = (0, 1)
2. If head and reverse: ($\mathbf{S_{AX}}$, $\mathbf{F_{AX}}$) = (K - 2, -1)
3. If tail and forward: ($\mathbf{S_{AX}}$, $\mathbf{F_{AX}}$) = (K - 1 - len(A), 1)
4. If tail and reverse: ($\mathbf{S_{AX}}$, $\mathbf{F_{AX}}$) = (len(A) - 1, -1)

where len(A) denotes the length of contig **A**.

We apply the inverse and composition rules to compute:

($\mathbf{S_{AB}}$, $\mathbf{F_{AB}}$) = ($\mathbf{F_{BX}}$ *($\mathbf{S_{AX}}$ - $\mathbf{S_{BX}}$), $\mathbf{F_{AX}}$*$\mathbf{F_{BX}}$).

Then there are four possible edges from A to B:

1. A-head to B-head then common K-1-mer is (head, reverse) for A and (head, forward) for B, so ($\mathbf{S_{AX}}$, $\mathbf{F_{AX}}$) = (K - 2, -1) and ($\mathbf{S_{BX}}$, $\mathbf{F_{BX}}$) = (0, 1) → ($\mathbf{S_{AB}}$, $\mathbf{F_{AB}}$) = (K - 2, -1)
2. A-head to B-tail then common K-1-mer is (head, reverse) for A and (tail, reverse) for B, so ($\mathbf{S_{AX}}$, $\mathbf{F_{AX}}$) = (K - 2, -1) and ($\mathbf{S_{BX}}$, $\mathbf{F_{BX}}$) = (len(B) - 1, -1) → ($\mathbf{S_{AB}}$, $\mathbf{F_{AB}}$) = (len(B) - K +1, -1)
3. A-tail to B-head then common K-1-mer is (tail, forward) for A and (head, forward) for B, so

(S_{AX}, F_{AX}) = (K - 1 - len(A), 1) and (S_{BX}, F_{BX}) = (0, 1) → (S_{AB}, F_{AB}) = (K - 1 - len(A), 1)

4. A-tail to B-tail then common K-1-mer is (tail, forward) for A and (tail, reverse) for B, so (S_{AX}, F_{AX}) = (K - 1 - len(A), 1) and (S_{BX}, F_{BX}) = (len(B) - 1, -1) → (S_{AB}, F_{AB}) = (len(A) + len(B) - K, -1)

We compose these maps along the forward (and backwards) path until we have traveled **EXTENT** base pairs. The result is that for all well-covered contigs, we can convert from coordinate reference frames of path-adjacent contigs. This is important in the third step, where we align well-mapped read pairs to each contig.

In the next step, we proceed one contig at a time and align all well-mapped read pairs. For each well-mapped read, we check if it has a good-ratio map to the present contig. If it does, we use that map. Otherwise, we check if the read has a good ratio map to any of the contigs in the de Bruijn graph extension of this contig. For each such alignment, we convert the map to the coordinates of the present contig using the inverse and composition rules. If all extension maps agree, we use that alignment. Otherwise, the read is unmapped. If either read in the pair is unmapped, we drop the read-pair.

Once all the well-mapped reads are aligned, we compute the consensus sequence over all covered positions. This results in a *coverage*, *maximal base*, and *maximal-base ratio* for each position in the alignment. Anywhere in the consensus where the coverage drops below **MIN_COVER** (10) and the maximal-base ratio drops below **MIN_RATIO** (0.95) is marked as a "disruption." If there is a disruption within the boundary of the initial contig, we do not extend the sequence. Otherwise, we extend the sequence with the maximal base up to the first disruptions outside of the initial boundary. We call the new sequence an ***extended contig***.

We then use the extension alignments from subpart 2 to co-align the extended contigs. If two contigs **A** and **B** are separated by a path in the de Bruijn graph shorter than **EXTENT** bases, we test if their extended contigs are compatible and non-trivial: they agree perfectly and overlap for **K** or more bases. We cluster together extended contigs according to this property and fuse the sequences together into ***final contigs***.

There is a post-processing step (6B) that cleans up the final contigs. First, it excludes any final contig that is a proper subsequence of another final contig. Second, it excludes all final contigs shorter than **MIN_ASSEMBLY_LENGTH** (1500).

Step 7: Unmask mutations

First, we load the unmutated (BS0) kmer counts we computed in Step 2. For each BS0 kmer and its reverse complement, we track its count in a dictionary called **bs0_count**. For both the kmer and its reverse complement, we convert every **C** to **T** and use this new sequence as a dictionary key to a list that contains that kmer, **ct_dict**. That way, **ct_dict** returns all possible unmutated preimages for any fully converted kmer.

Then, for each final contig, we list its consecutive kmers. For each kmer, **x**, we apply a full **C-to-U** conversion and look up the resulting kmer in **ct_dict[convert(x)]**. For each possible pre-image, we test if it is compatible with conversion to **x**. In particular, no **T** in the preimage converts to a **C** in **x**. For each compatible preimage **y**, we align **y** to the final contig with a weight of **bs0_count[y]**. We repeat this for every kmer in the final contig and then compute the consensus sequence over the weighted alignments. Wherever the maximal base ratio exceeds **MIN_MAX_RATIO** (0.99), we use the maximal base, keeping the existing base otherwise. In this way, positions that are truly polymorphic for C-to-U in the unmutated sequence data remain uncorrected. We call the resulting sequence the **unmasked contig**.

The unmasking method is strand-specific, in that we assume that the final contig is the same sequence that was subject to mutation. For that reason, we apply the same unmasking method to the

reverse complement of the final contig. Whichever of the two (final contig or reverse complement of final contig) has the most weighted pre-image maps we select as the correct orientation for the final contig.

At this stage, we can also estimate the mutation rate for each final contig by identifying the rate at which **C** positions in the unmasked contig correspond to **T** positions in the final contig. We restrict downstream analysis to those contigs with a mutation rate of at least **MUT_RATE_MIN** (0.25).

Step 8: Align and phase

The alignment and phasing take place in a single function but involve multiple subcomponents. The first part aligns each unmasked contig against the longest unmasked contig, which we designate as the ***reference contig***. The second part determines a set of polymorphic positions in the common alignment. The third component uses an error and mutation model to identify an optimal set of haplotypes to explain the polymorphism data. When the data size is small, this can be computed explicitly. For more complex problems, we resort to simulated annealing. The present version requires an input specifying the number of haplotypes (**NUMBER_OF_HAPLOTYPES** = 2, 4, depending on application). The fourth step assigns each unmasked contig to a haplotype (or "unassigned" if insufficient information.)

In the first step, we align all unmasked contigs to the longest unmasked contig. We use the Needleman-Wunsch algorithm for pairwise alignment which we implemented in C accessible through python. To enable a free gap at the beginning of the alignment, we modify the trace to allow free movement along the left and top edges of the matrix. To enable a free gap at the end of the alignment, we create a corridor from the best answer on the bottom or the right edge of the matrix to the bottom right corner, where the trace-back begins. We align both the unmasked contig and its reverse complement, selecting the alignment with the best score. If the unmasked contig was reversed for the alignment, we record this information since this matters later with respect to C/T and G/A polymorphisms.

After aligning all **N** contigs to the reference contig, we identify a set of **P** positions that may vary between haplotypes. These are positions where the ratio of the maximum base (computed over the **N** contigs) is below **BASE_RATIO_CUTOFF** (0.85). We generate the **het_data** matrix with shape (**N**, **P**) recording the base observations at those positions. For each sequence, we also record in the vector **reverse** if the mutations in the sequence were C->T (false) or G->A (true).

To compute emission probabilities, we treat each position **p** as bi-allelic, choosing the two bases with maximum frequency and then sorting them alphabetically into a generic **A/B** locus (i.e. C/T poly C=A allele, T=B allele). For a given error rate (**err**) and mutation rate (**mut**), we compute the probability of the observation in **het_data[n, p]**, assuming true allele is **A** or **B**. For most cases, we compute the probability as:

emit_PN2[p, n, A=0] = (het_data[n,p] == A)*(1-**err**) + (het_data[n, p] == B)*(**err**)

emit_PN2[p, n, B=1] = (het_data[n,p] == B)*(1-**err**) + (het_data[n, p] == A)*(**err**)

The two special cases are:

A=C, B=T and reverse[n] = False, then emitting the A allele (C) depends on not mutating:

emit_PN2[p, n, A=0] = (het_data[n,p] == A)*(1-**mut**) + (het_data[n, p] == B)*(**err**)

Similarly, for A=A, B=G and reverse[n] = True, then emitting the B allele (G) also depends on not mutating:

emit_PN2[p, n, B=1] = (het_data[n,p] == B)*(1-**mut**) + (het_data[n, p] == A)*(**err**)

All unset entries (where the observations are neither A nor B) are set to a probability of 1.

We define a haplotype **h** as a vector of **p** binary choices (0 = A allele, 1 = B allele). The probability of a given sequence observation **het_data[n, :]** is the product of the emission vector specified by the haplotype choice:

$$P(het\_data[n,:]|h) = \prod_{p} emit\_PN2[p,n,h[p]]$$

For **H** haplotypes (K = 2 for diploid), $\mathbf{h_1 \ldots h_K}$ we define

$$P(het\_data[n,:]|H) = \max_{k} P(het\_data[n,:]|h_k)$$

And the total probability of all the data are:

$$P(het\_data|H) = \prod_{n} P(het\_data[n,:]|H)$$

For cases where the number of haplotypes sets to explore is manageable, we compute over the set of all possible **H** (2^{K*P} for K haplotypes and P positions). Otherwise, we start with a random choice of **H** and apply simulated annealing (SA) to search for an optimal haplotype configuration.

Briefly, the simulated annealing algorithm works as follows. We first build a random set of **K** haplotypes, **H_KP** as a random binary matrix with shape (K, P). Our moves in the simulated annealing algorithm involve flipping a single bit in the haplotype matrix, **H_KP**. We determine the maximum change for any single move and set that to our starting temperature **T**. The consequence of this is that when the algorithm starts, the biggest possible move will be accepted. The SA process has three steps:

1. Propose a move, flipping a single bit in the haplotype matrix.
2. Compute the change in probability induced by the move.
3. If the new probability is higher than the old, we accept the move. If not, we accept the move with probability exp((new_prob – old_prob) / temperature).

We propose **STEPS_PER_TEMP** (1000) moves per temperature and then reduce the temperature **T** to **ALPHA*T** (**ALPHA**= 0.95) for the next set of **STEPS_PER_TEMP**. This will continue for up to **MAX_TEMP_STEPS** (10000) or until no moves are accepted for **MAX_STUCK** (100) successive temperatures.

Finally, we assume a uniform prior probability for the haplotypes and compute the posterior probability that each unmasked contig originated from each haplotype. The posterior probability that the contig ***n*** derives from the haplotype ***k*** is given by:

$$P(h_k \mid het\_data[n,:]) = \frac{P(het\_data[n,:]|h_k)}{\sum_k P(het\_data[n,:]|h_k)}$$

If the posterior probability of the most likely haplotype exceeds 1 - **MIN_HAPLO_CONFIDENCE** (0.001), then we assign the contig to the haplotype.

Step 9: Compute consensus over haplotypes

In the final step, we compute the consensus sequence for each haplotype in turn. This requires two steps: first assigning a common alignment to all the contigs in the haplotype and then determining the most likely sequence together with a confidence score per position.

First, we select the longest unmasked contig to serve as the initial reference sequence for the haplotype. We then use the Needleman-Wunsch pairwise alignment described above to align all the remaining contigs to this initial reference sequence with one key difference. Previously, the contigs came

from different alleles and are expected to vary by insertion and deletion polymorphisms. In this instance, however, we expect indels and base errors to represent rare event resulting from polymerase error or somatic variation. For this reason, if the initial alignment results in a common indel event, that indel is introduced back into the reference sequence and we repeat the process until no such indels are encountered. For **N** templates and **P** positions in the reference template, we obtain an **N**-by-**P** matrix, **align,** with the base calls (or gaps) of the templates as aligned to the reference.

To determine the consensus sequence, we apply a probabilistic model similar to that in step 8. If a position is unmasked, then the observation probability is entirely driven by error:

$$P(align[n,p] \mid X) = \begin{cases} 1 - err & if\ align[n,p] = X \\ err & if\ align[n,p] \neq X \end{cases}$$

If, however, the position is not unmasked then the probability also depends on the strand of the conversion and the particular base pair. For positions that are not unmasked and are C-to-U converted in the present alignment:

$$P(align[n,p] \mid C) = \begin{cases} 1 - mut & if\ align[n,p] = C \\ mut & if\ align[n,p] = T \\ err & otherwise \end{cases}$$

And likewise, for positions that are not unmasked and G-to-A converted:

$$P(align[n,p] \mid G) = \begin{cases} 1 - mut & if\ align[n,p] = G \\ mut & if\ align[n,p] = A \\ err & otherwise \end{cases}$$

Then the probability for each position is given by:

$$P(align[:,p] \mid X) = \prod_n P(align[n,p] \mid X)$$

And apply Bayes formula and uniform prior to obtain

$$P(X \mid align[:,p]) = \frac{P(align[:,p] \mid \mathrm{X})}{\sum_Y P(align[:,p] \mid \mathrm{Y})}$$

We report the base X with the highest probability and return the log10 transformed Phred score of

$$score = -10 * \log 10(1 - P(X \mid align[:,p]))$$

This procedure is repeated for each haplotype in turn and the final answer is returned in the file **haplotype_assemblies.fastq**.

Measuring PCR error rates

Recombination

Using a single bottom strand library from region 3, we have 1,313 extended contigs that are greater than 1.5 KB in length. There are 14 SNV positions over the 10.5 KB stretch that delineate the two different haplotypes. For each contig, we compute the probability that the observed sequence derives from haplotype 1 or haplotype 2 (see Supplementary Figure 4). Nearly every assigned contig is in uniform agreement with one of the two haplotypes. Almost all of the unassigned contigs are too short, having only one or two SNVs which is insufficient to confidently call phase. There is one exception, marked by the

arrow in Supplementary Figure 4 – an unassigned contig that, on inspection, shows seven SNVs, the first four from one haplotype and the last three from the other.

SNV and indel error

Using the haplotype sequence as a reference, we used the Needleman-Wunch algorithm to align each unmasked contig to its assigned haplotype. We recorded all differences in point mutations (PCR SNV errors) and gaps in alignment (PCR indels). We found that the rate of PCR SNVs is ~7.4e-4 per base and the indel error rate is ~7.7e-5. Upon examination, 68% of indels are at microsatellites (mono or di repeats) and 92% are at microsatellites when the genome is fully converted.
